# The relationship between the expression of tumor matrix-metalloproteinase and the characteristics of magnetic resonance imaging of human gliomas

**DOI:** 10.1016/S1674-8301(10)60020-6

**Published:** 2010-03

**Authors:** Lihua Liu, Ming Zhang, Yuan Wang, Min Li

**Affiliations:** Center of Image, the First Affilliated Hospital to Medical College of Xi'an Jiaotong University, Xi'an, Shaanxi Province 710061,China

**Keywords:** matrix-metalloproteinase, magnetic resonance imaging, glioma

## Abstract

**Objective:**

To investigate the relationship between the expression level of matrix-metalloproteinases (MMPs) with the pathological grades and MRI characteristics of human gliomas.

**Methods:**

Prior pre- and post-contrast enhancement MRI was performed on 31 patients with gliomas, which were confirmed by post-operational pathology. The expression of MMP-2 and MMP-9 were determined by immunohistochemical staining in both a low grading group (grades I and II, *n* = 20) and high grading group (grades III and IV, *n* = 11).

**Results:**

Compared to the low grading group, the expression levels of MMP-2, MMP-9, as well as the tumor edema index (EI), enhanced percentage (EP) and maximum diameters were significantly greater in the high grading group. MMP-2 and MMP-9 expression were correlated with the tumor EI, EP and the maximum diameters. There were no differences in MMP-2 and MMP-9 expression between the unclear border definition group and the clear border definition group, whereas the MMPs expression levels were greater in the heterogeneous signal group than in the homogeneous signal group.

**Conclusion:**

The expression level of MMPs is correlated with the invasion ability of human gliomas. The MRI parameters, such as tumor EI, EP, maximum diameter, and signal heterogeneity technically reflect the expression level of MMPs, and can be used to estimate the tumor's malignant behavior, thus providing the guidance for clinical therapies.

## INTRODUCTION

Glioma is one of the most commonly occurring primary tumors in the Central Nervous System (CNS). Current therapies produce very few optimal effects due to the invasive growth feature of the tumor. MRI is a strong tool to aid in defining the glioma's pathological grade and related invasive capability, and by providing therapeutic guidance in clinical treatment. Many studies have shown that the ability of a glioma to invade tissue is closely correlated with the expression levels of matrix-metalloproteinases (MMPs). The current study investigated the relationship between the expression levels of MMPs and the MRI characteristics as well as the pathological grade of human gliomas, thus providing guidance in selecting the best therapy and evaluating the prognosis after clinical treatment.

## MATERIALS AND METHODS

### Clinical Information

The human glioma samples were obtained from 31 glioma patients who had their surgeries between July 2003 and April 2005 in Xi'an Jiaotong University First Affiliated Hospital. No radiotherapy or chemotherapy was given to any patient before the operation. The study involved 16 male and 15 female patients, between the ages of 15-71, and the average age was 44.7±16.2 years. The most common clinical symptoms and complaints among the patients were headache(*n* = 17), epileptic seizures (*n* = 7), disturbances in the feelings and movements of limb (*n* = 5), and psychosis (*n* = 2). The patients were divided into 2 groups, low grading group (*n* = 20, grade I and grade II) and high grading group (*n* = 11, grade III and grade IV) according to the 2007 WHOCNS tumor classification.

### MRI scanning

All MR scans performed prior to the operations were conducted on a 1.5-T MR Imaging System (Gyroscan Intra, Philips) with the Head Coil and SE sequence. Pre- and postcontrast enhancement scans were conducted in all patients before their operations. The glioma diagnoses were confirmed by pathological analysis after surgery. The MRI regular scanning included sagittal T1WI, transverse T1WI and T2WI, and the MRI enhancement scanning were fulfilled by Gd DAPA in sagittal and transverse T1WI. The dosage of Gd DAPA was 0.1 mmol/kg and the injection was completed within 1-2 min. The following T1WI scan parameters were applied: TR/TE = 431/11 ms, T2WI sequence TR/TE = 4850/120 ms, FOV = 24 cm×24 cm, matrix = 256×256, NEX = 2, bandwidth = 12.5 kHz, slice thickness = 5 mm, and spacing = 1 mm. The same parameters were used in the enhancement scanning.

### Immunohistochemistry

The pathological specimens were fixed and embedded in paraffin and then sectioned. After dewaxing, section endogenous peroxidase was blocked with 3% H_2_O_2_ for 10 min, and incubated with mouse anti-human MMP-2 or MMP-9 primary antibody (Boshide Inc., China) at 4°C overnight. Subsequently, the sections were incubated with the goat anti-mouse secondary antibody at room temperature for 30 min. After being stained with DAB and counterstained with haematoxylin, the sections were studied by light microscopy. Some previously obtained sections showing positive staining were used as positive controls. In negative control sections the primary antibody was omitted.

### Analysis and evaluation of MMPs expression

The expression of MMPs was analyzed under a high-resolution light microscope, based on the immunohistochemical staining results. Positive cells were defined as cells with brown or brown-yellow staining in the cytoplasm. The distribution and expression levels of MMPs were then quantified. The average expression level of MMPs (L1) was calculated by the equation: L1 = (numbers of positive staining cells / total numbers of counted cells) x 100%.

### MRI data analysis

#### Measurement of Edema Index (EI)

The EI was determined as previously described by Inamura *et al*[1]. The maximum diameter and the maximum length of the tumor were evaluated in enhanced transverse plane images, and the maximum height was measured in enhanced sagittal plane images. The volume of the tumor was calculated by multiplying these three maximum numbers. The volume of edema was measured at the T2WI sequence by the same method. EI = volume of edema/volume of tumor.

#### Measurement of enhancement

The regions of interest (ROI) was selected on the maximum layer of the tumor, which was approximately 20-30 mm^2^. Intensities of the signals were measured at T1WI or T1WI enhancement sequence, respectively. The mean value obtained subsequently was the intensity of the signals before and after the enhancement scan. The Enhance Percentage (EP) that was used to assess the enhancement of tumors was the difference between the intensities of the signals before and after the enhancement scan divided by the intensity of the signals before the scan.

The same experimental methods and standards were used in all patients. The ROI was selected and measured consistently.

#### Other data

Using enhanced transverse T1WI images, the maximum diameters were measured at the layers with maximum tumor dimensions.

All MRI scans were evaluated and verified by two experienced radiologists.

### Statistical analysis

All measurement data were expressed as mean±SD, and two independent samples t test was used to compare the differences of two independent samples. Pearson's test was used in correlation analyses. The P value reported was two-sided and values of *P* < 0.05 were considered statistically significant. All analyses were performed using the SPSS software (Version12.0, SPSS Inc, USA).

## RESULTS

### Immunohistochemical results

**Fig.1:**
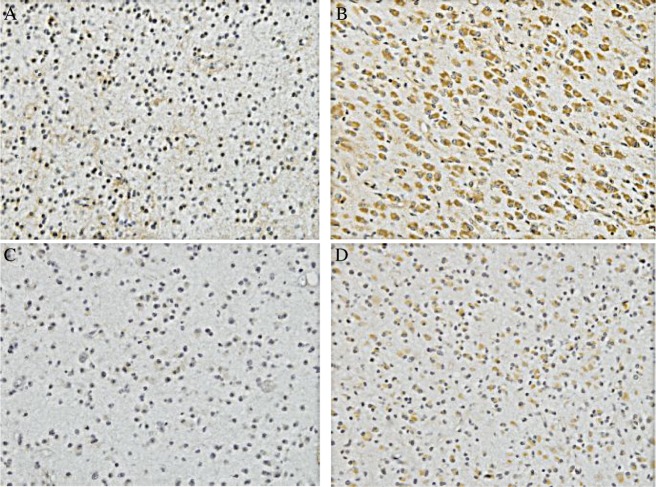
Immunohistochemical results of the expression of MMP-2 (A and B)and MMP-9 (C and D) A: Expression of MMP-2 in the low grading glioma (×100). B: Expression of MMP-2 in the high grading glioma (×100). C: Expression of MMP-9 in the low grading glioma (×100). D: Expression of MMP-9 in the high grading glioma (×100). Fewer positive cells with weak staining were observed in the low grading glioma (A and C). Compared to the low grading group, more positively stained cells with dark-brown granules were observed in the high grading gliomas (B and D).

The expression of MMP-2 (***Fig.1A and 1B***) and MMP-9 (***Fig.1C and 1D***) in the glioma specimens was observed in the tumor cellular cytoplasm, the vascular endothelial cells and the basement membranes. Small numbers of positive cells with weak staining were observed in the low grading gliomas. Compared to the low grading group, in the high grading gliomas there were more positively stained cells with dark-brown granules and the numbers of blood-vessels increased (***Fig.1***). In addition, the positively stained cells were locally nested and their distribution showed the characteristic of “bigger spots”. The strong staining of MMPs was located mainly in the active proliferation and invasion areas. In the low grading gliomas, quantitative analysis revealed the mean expression levels of MMP-2 and MMP-9 staining were 16.38±8.43% and 12.09±7.98%, respectively, compared to those of 60.47±21.45% and 41.03±14.00% in the high grading gliomas (***Table 1***). Thus, MMP-2 and MMP-9 expression was significantly different between the low grading group and the high grading group (*P* < 0.05).

**Table 1:** Relationships between the pathological grade and the glioma MMPs expression, EI and EP values and the maximum tumor diameter

	Low grading glioma (*n* = 20)	High grading gliomas (*n* = 11)	*t*	*p*
MMP-2(%)	16.38 ± 8.43	60.47 ± 21.45	8.200	< 0.05
MMP-9(%)	12.09 ± 7.98	41.03 ± 14.00	6.312	< 0.05
EI	1.21 ± 0.67	6.67 ± 5.55	4.405	< 0.05
EP	4.66 ± 4.61	33.86 ± 20.08	6.286	< 0.05
Maximum diameter (mm)	34.02 ± 10.51	53.19 ± 9.97	5.026	< 0.05

### MRI

**Fig.2:**
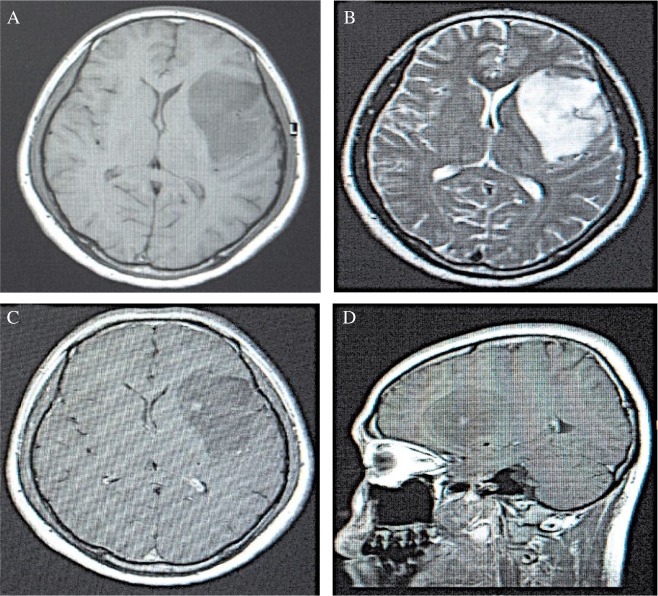
The MRI scanning result of a low grade glioma. A: A homogeneous and long T1 signal with clear border was seen in the T1WI sequence at the transverse plane in the left frontal lobe. The lateral ventricle was compressed. B: A homogeneous and long T2 signal with clear border was observed in the transverse plane T2WI sequence in the left frontal lobe, with lateral ventricle compression. C: A T1WI enhancement scanning at the transverse plane showed a slightly irregular enhancement. D: A T1WI enhancement scanning at the sagittal plane showed a slightly irregular enhancement.

**Fig.3:**
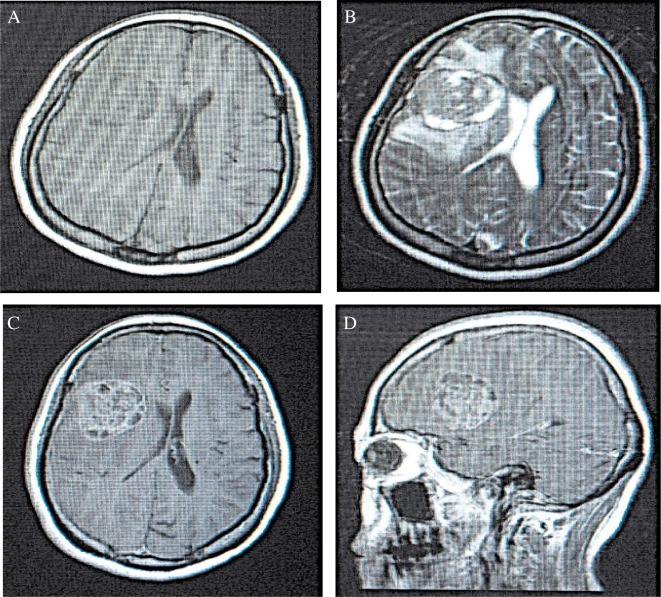
The MRI of a high grade glioma. A: A heterogeneous and slightly long T1 signal with unclear border was detected in the T1WI sequence at the transverse plane in the left frontal lobe. B: A heterogeneous and slightly long T2 signal with unclear border was observed in the T2WI sequence at the transverse plane in the left frontal lobe. An edema band existed around the tumor. C: A T1WI enhancement scan at the transverse plane showed a wreath-like and heterogeneous enhancement. D: A T1WI enhancement scanning at the sagittal plane showed a wreath-like and heterogeneous enhancement.

In the low grading gliomas, lower or slightly lower signals were observed in the T1WI sequence, in which the majority showed a homogeneity feature. In the T2WI sequence, higher or slightly higher signals were observed, which were mainly homogeneous with clear borders. In addition, the signals accompanied by rare bleeding were generally not enhanced or only slightly enhanced (***Fig.2***). Signals in the T1WI sequence in the high grading group were heterogeneously low and equi-signals, in which few showed the low and high heterogeneous distribution. In the T2WI sequence, heterogeneity of equi- and high signals with unclear borders accompanied by necrosis and bleeding were observed. Furthermore, obviously heterogeneous enhancements were seen in most of the high grading gliomas (***Fig.3***). In some patients, tumors invaded the corpus callosum, crossing the midline and reaching the contra-lateral cerebral hemisphere. Multiple tissues or organs were invaded in rare cases. Quantitive results showed that EI was 1.21±0.67, EP was 4.66±4.61, and the maximum diameter was 34.02±10.51 mm in the low grading gliomas. The corresponding values in the high grading gliomas were: EI = 6.67±5.55, EP = 33.86±20.08, and the maximum diameter = 53.19±9.97 mm, respectively.

### Relationship between the pathological grade and the MMPs expression, EI, EP value, and maximum tumor diameter.

A *t*-test analysis showed that the MMPs expression, EI or EP value and the maximum diameter significantly differed between the different groups. In the high grading group, the MMP-2 and MMP-9 expressions were significantly higher than in the low grading group (*P* < 0.05). Similarly, the values of EI, EP, and the maximum diameter were significantly higher than those in the low grading glioma group (*P* < 0.05).

### Correlation between the levels of MMPs expression and EI, EP and the maximum diameter of tumors

**Fig. 4:**
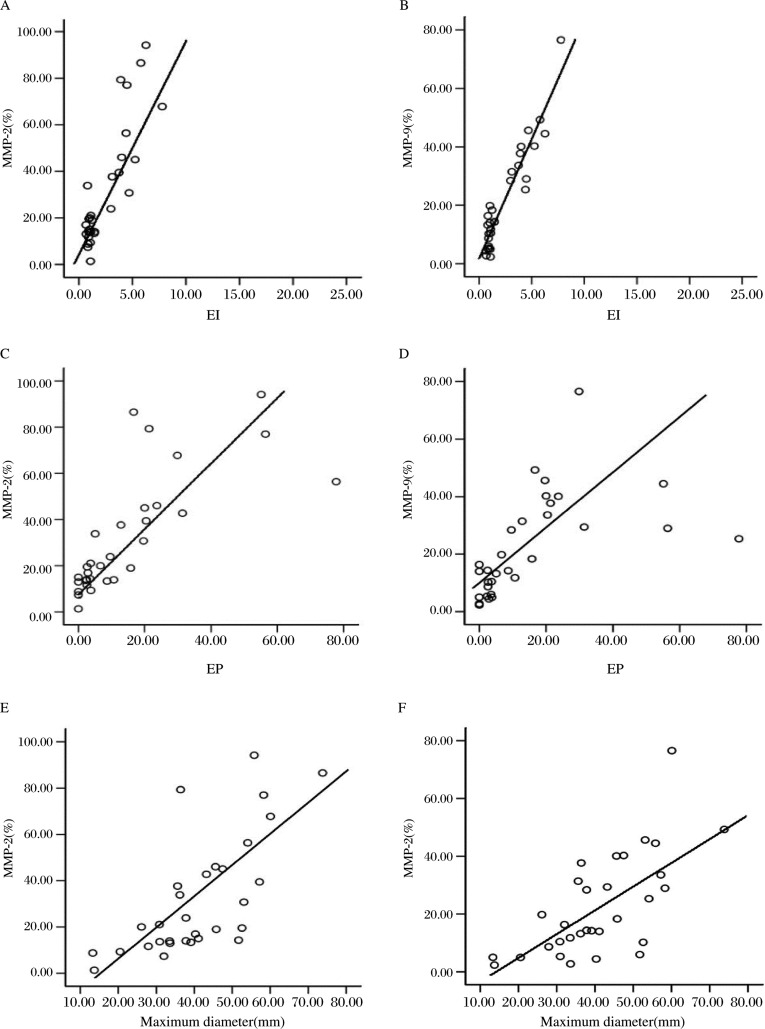
The correlation between the expression of MMP-2, MMP-9 and EI (A and B), EP (C and D) and the maximum diameter (E and F) of tumor.

The MMP-2 (*r* = 0.480, *p* < 0.05) or MMP-9 (*r* = 0.516, *p* < 0.05) expression showed a positive correlation with the corresponding EI (***Fig. 4A and 4B***)

The MMP-2 (*r* = 0.754, *p* < 0.05) or MMP-9 (*r* = 0.554, *p* < 0.05) expression showed a positive correlation with the corresponding EP (***Fig. 4C and 4D***)

The MMP-2 (*r* = 0.700, *p* < 0.05) or MMP-9 (*r* = 0.676, *p* < 0.05) expression showed a positive correlation with the corresponding maximum diameter (***Fig. 4E and 4F***)

### Assessments of the correlation between the MMPs expression and other MRI parameters

Based on the clarity of borders and the degree of signal homogeneity, the gliomas investigated in the current study were re-divided into two groups with unclear/clear borders, or two groups with heterogeneous/homogeneous signals. The mean ratio of MMP-2 (MMP-9) expression in the unclear border group was 31.67±26.42% (20.02±14.85%) and was 32.67±25.45% (26.62±21.84%) in the clear border group. In addition, the mean ratio of MMP-2 or MMP-9 expression in the heterogeneous signal group was 37.84±27.73% or 26.28±18.48%, and in the homogeneous group the values were 17.82±11.41% and 12.77±9.84%. The *t*-test showed a significant difference in both the MMP-2 and MMP-9 expression between the heterogeneous and homogeneous signal groups, but there were no differences between the unclear border group and the clear border group (***Table 2***).

**Table 2:** MMPs expression and other MRI characteristics

	MMP-2	MMP-9	t	*P*
Clear border group (*n* = 14)	32.67 ± 25.45%	26.26 ± 21.48%	0.103	> 0.05
Unclear border group (*n* = 17)	31.67 ± 26.42%	20.02 ± 14.85%		
Homogeneous signal group (*n* = 18)	17.82 ± 11.41%	12.77 ± 9.84%	2.848	< 0.05
Heterogeneous signal group (*n* = 13)	37.84 ± 27.73%	26.28 ± 18.48%		

## DISCUSSION

Previous studies revealed that, among the information provided by MRI, EP, EI, tumor size, border definition, signal homo- or heterogeneity, space-occupying effect, and midline shift, are positively correlated with the pathological grade of gliomas[2]–[4]. Importantly, EP and EI have been shown to exhibit the strongest correlations. However, the underlying molecular mechanisms for such correlations are still unclear.

Our study investigated the expression of MMPs in human gliomas by using an immunohistochemical staining method. Our results illustrated that the expression levels of MMP-2 and MMP-9 are associated with the pathological grades of the tumor[5]–[6], providing strong evidence that MMPs expression can be used as a criterion to determine the malignant phenotypes of gliomas.

Results from the present study demonstrated that: ①brain edema or space-occupying effect rarely occur in low grading tumors, whereas moderate or severe brain edema often appears in high grading gliomas accompanied by an obvious space-occupying effect, suggesting the severity of edema is closely related to the expression level of MMPs. ②MRI results also provide diagnostic evidence of a low grading glioma based on the lack of apparent enhancement at the interface of brain and tumor. In the higher pathological grades of gliomas the interface of the brain and tumor were often shown to be irregular, heterogeneous and gradually intensified. ③Although our results showed that the size of a tumor is another factor relating to the MMPs expression, we still cannot speculate on the malignancy of the tumors by tumor size due to the fact that the tumor size is also determined by tumor localization, initiation and duration of the tumor growth, and other factors. ④The expression of the MMP-2 and MMP-9 were significantly different between the heterogeneous and homogeneous signal groups, but were not different between the unclear border group and the clear border group. These findings suggest that some of the MRI features in the current study might be associated with the expression of MMPs.

MMPs belong to a family of proteolytic enzymes that play an important role in tumor invasion, matrix degradation, blood-brain barrier (BBB) destruction and tumor angiogenesis[7]–[10].

The expression levels of MMPs are increased in the higher pathological grades of gliomas[11], leading to the opening of the BBB by degrading capillary beds enriched in basement membranes. As a result, at the early stage the permeability of the BBB increases, promoting the development of vasogenic cerebral edema. Meanwhile, inflammatory cells enter the tumor tissues via the injured BBB, which leads to a more serious cerebral damage (cytotoxic cerebral edema), by releasing inflammatory mediators[12],[13]. Accumulated evidence demonstrates that MMP-2 up-regulation contributes to the early opening of BBB[14], whereas the later stage opening is related to MMP-9 expression[15].

In addition, MMPs play an essential role in regulating the formation of tumor blood vessels during tumor invasion[16],[17]. The structure of newly-generated blood vessels is incomplete, which leads to the leakage of plasma and large molecules into the intercellular space, resulting in the development of edema around the tumors. Moreover, up-regulation of MMPs accelerates the angiogenesis of tumors, which appears to be related to the MRI enhancement results[18]–[22].

In high grading gliomas, MMPs up-regulation enhances the capability of tumor invasion which accelerates the development of cystic change, necrosis, calcification and hemosiderosis in tumor cells[23],[24]. The complicated cellular components of high grading tumors parallel the heterogeneous signals in MRI scanning results, demonstrating the heteromorphism of the tumor tissues[25]. Invasive growth is the characteristic of gliomas, even in low grade gliomas, and the diffusive, invasive growth of tumor cells is apparent, indicating that local invasion is a common feature of gliomas[26]. Distributions of tumor cells that are located in the white matter and gray matter of the cerebral hemispheres are mostly diffuse, and mainly include astrocytes with unclear borders. Therefore, the clarity of border indicated in an MRI image cannot be used as a marker to diagnose the malignancy of tumor.

In conclusion, the expression level of MMPs is correlated with the invasion ability of human gliomas[27]–[30]. The MRI parameters, such as tumor EI, EP, maximum diameters, and signal heterogeneity, can be used to reflect the expression level of MMPs and estimate the tumor's malignant behavior, thus providing the guidance for clinical therapies.

## References

[1] Inamura T, Nishio S, Takeshita I, Fujiwara S, Fukui M (1992). Peritumoral brain edema in meningiomas-influence of vascular supply on its development. Neurosurg.

[2] Chen CR, Liao WH, Chen CY (2004). Correlation between peritumoral edema on MRI, VEGF expression, MVD and pathological grading in astrocytomas. China J Modern Med.

[3] Galanaud D, Nicoli F, Fur YL, Guye M, Ranjeva JP, Gouny SC (2003). Multimodal magnetic resonance imaging of the central nervous system. Biochimie.

[4] Rees J, Watt H, Jäger HR, Benton C, Tozer D, Tofts P (2009). Volumes and growth rates of untreated adult low-grade gliomas indicate risk of early malignant transformation. Eur J Radiol.

[5] Their M, Roeb E, Breuer B, Bayer T, Halfter H, Weis J (2000). Expression of matrix metalloproteinase-2 in glial and neuronal tumor cell lines: inverse correlation with proliferation rate. Cancer Lett.

[6] T Turpeenniemi-Hujanen (2005). Gelatinases (MMP-2 and -9) and their natural inhibitors as prognostic indicators in solid cancers. Biochimie.

[7] Guo P, Imanishi Y, Cackowski FC (2005). Up-regulation of angiopoientin-2, matrixmentalloprotease-2, membrane type 1 mentalloprotease, and laminin 5 gamma 2 correlates with the invasiveness of human glioma. Am J Pathol.

[8] Kanazawa R, Yoshida D, Takahashi H, Sugisaki Y, Suzuki S, Teramoto A (2004). Drug-induced apoptosis by a matrix metalloproteinase inhibitor, SI-27 on human malignant gliomas cell lines; in vitro study. J Neurooncol.

[9] Yakahaski M, Fukami S, Iwata N (2002). In vivo glioma growth requires host-derived matrix metalloproteinase 2 for maintenance of angioarchitecture. Pharmacol Res.

[10] Tate MC, Aghi MK (2009). Biology of angiogenesis and invasion in glioma. Neurotherapeutics.

[11] Dai B, Kang SH, Gong W, Liu M, Aldape KD, Sawaya R (2007). Aberrant FoxM1B expression increases matrix metalloproteinase-2 transcription and enhances the invasion of glioma cells. Oncogene.

[12] Nordqvist AC, Smurawa H, Mathiesen T (2001). Expression of matrixmetalloproteinase 2and 9 in meningiomas associated with different degrees of brain invasiveness and edema. J Neurosurg.

[13] Yong VW, Forsyth PA, Bell R, Krekoski CA, Edward DR (1998). Matrix metalloproteinases and diseases of the CNS. Trends Neurosci.

[14] Forsyth PA, Wong H, Laing TD, Rewcastle NB, Morris DG, Muzik H (1999). Gelatinase-A(MMP-2), gelatinase-B(MMP-9) and membrane type matrix metalloproteinase-1(MT1-MMP) are involved in different aspects of the pathophsiology of malignant gliomas. Br J Cancer.

[15] Jiang CZ, Lin ZX, Chen ZB (2003). The influence of MMP-2 and MMP-9 on blood-tumor barrier of invasive microecosystem in human glioma. Chin J Neuroimmunol Neurol.

[16] Rao JS, Gondi C, Chetty C, Chittitivelu S, Joseph PA, Lakka SS (2005). Inhibition of invasion, angiogenesis, tumor growth, and metastasis by adenovirus mediated transfer of antisense uPAR and MMP-9 in non-small cell lung cancer cells. Mol Cancer Therap.

[17] Yamamoto M, Mohanam S, Sawaya R, Fuller GN, Seiki M, Sato H (1996). Differential expression of membrane-type matrix metalloproteinase and its correlation with gelatinase A activation in human malignant brain tumors in vivo and in vitro. Cancer Res.

[18] Roberts HC, Roberts TP, Brasch RC, Brasch, Dillon WP (2000). Quantitative measurement of microvascular permeability in human brain tumors achieved using dynamic contrast enhanced MRI imaging: correlation with histologic grade. Am J Neuroradiol.

[19] Earnest F, Kelly PJ, Scheithauer BW, Kall BA, Cascino TL, Ehman RL (1998). Cerebral astrocytomas: histopathologic correlation of MR and CT contrast enhancement with stereotactic biopsy. Radiol.

[20] Watanabe M, Tanaka R, Takeda N (1992). Magnetic resonance imaging and histopathology of cerebral gliomas. Neuroradiol.

[21] Weiss N, Miller F, Cazaubon S, Couraud PO (2009). The blood-brain barrier in brain homeostasis and neurological diseases. Biochim Biophys Acta.

[22] Wong LH, Prawira A, Kaye AH, Hovens CM (2009). . Tumour angiogenesis: Its mechanism and therapeutic implications in malignant gliomas. J Clin Neurosci.

[23] Park JM, Kim A, Oh JH, Chung AS (2007). Methylseleninic acid inhibits PMA-stimulated pro-MMP-2 activation mediated by MT1-MMP expression and further tumor invasion through suppression of NF-кB activation. Carcinogenesis.

[24] Kurukawa K, Kumon Y, Harada H, Kohno S, Nagato S, Teraoka M (2006). PTEN gene transfer suppresses the invasive potential of human malignant gliomas by regulating cell invasion-related molecules. Int J Oncol.

[25] Steen RG (1992). Edema and tumor perfusion: characterization by quantitative 1H MR imaging. Am J Roentgenol.

[26] Bagley LJ, Grossman RI, Judy KD, Curtis M, Loevner LA, Polansky M (1997). Gliomas: correlation of magnetic susceptibility artifact with histologic grade. Radiol.

[27] Arribas J (2005). Matrix metalloproteases and tumor invasion. N Eng J Med.

[28] Demuth T, Berens ME (2004). Molecular mechanisms of glioma cell migration and invasion. J Neuro-Oncol.

[29] Nakada M, Nakada S, Demuth T, Tran NL, Hoelzinger DB, Berens ME (2007). Molecular targets of glioma invasion. Cell Molec Life Sc.

[30] Nuttall RK, Pennington CJ, Taplin J, Wheal Alison, Yong VW, Forsyth PA (2003). Elevated membrane-type matrix metalloproteinases in gliomas revealed by profiling proteases and inhibitors in human cancer cells. Molec Cancer Res.

